# A comparison of the innate flexibilities of six chains in F_1_-ATPase with identical secondary and tertiary folds; 3 active enzymes and 3 structural proteins

**DOI:** 10.1063/1.4967226

**Published:** 2016-11-04

**Authors:** Monique M. Tirion

**Affiliations:** Physics Department, Clarkson University, Potsdam, New York 13699-5820, USA

## Abstract

The *α* and *β* subunits comprising the hexameric assembly of F1-ATPase share a high degree of structural identity, though low primary identity. Each subunit binds nucleotide in similar pockets, yet only *β* subunits are catalytically active. Why? We re-examine their internal symmetry axes and observe interesting differences. Dividing each chain into an N-terminal head region, a C-terminal foot region, and a central torso, we observe (1) that while the foot and head regions in all chains obtain high and similar mobility, the torsos obtain different mobility profiles, with the *β* subunits exhibiting a higher motility compared to the *α* subunits, a trend supported by the crystallographic B-factors. The *β* subunits have greater torso mobility by having fewer distributed, nonlocal packing interactions providing a spacious and soft connectivity and offsetting the resultant softness with local stiffness elements, including an additional *β* sheet. (2) A loop near the nucleotide binding-domain of the *β* subunits, absent in the *α* subunits, swings to create a large variation in the occlusion of the nucleotide binding region. (3) A combination of the softest three eigenmodes significantly reduces the root mean square difference between the open and closed conformations of the *β* subunits. (4) Comparisons of computed and observed crystallographic B-factors suggest a suppression of a particular symmetry axis in an *α* subunit. (5) Unexpectedly, the soft intra-monomer oscillations pertain to distortions that do not create inter-monomer steric clashes in the assembly, suggesting that structural optimization of the assembly evolved at all levels of complexity.

## I. INTRODUCTION

### A. Overview of hexameric F_1_-ATPase

ATP synthases exploit ion gradients generated during electron transport reactions at cell interfaces to phosphorylate ADP and replenish the cell's supply of ATP. Mild salt treatments dissociate ATP synthases into two fractions: a membrane-embedded F_*o*_ portion and a soluble, hydrophilic F_1_ portion (for reviews, see Refs. 1–3). In the intact enzyme, the F_*o*_ portion links an ionic gradient to a mechanical rotation, while the F_1_ portion channels the rotary motion to the synthesis reaction. The dissociated F_1_ portion lacks the capacity to generate ATP; however, it does function as an ATPase, hydrolyzing ATP in the presence of ATP, ADP, and phosphate, P_*i*_. The isolated F_1_ complex consists of five different protein chains with stoichiometries of *α*_3_*β*_3_*γ*_1_*δ*_1_*ϵ*_1_ and mass ratios 55, 51, 31, 15, and 6 kD. F_1_ fractions obtained from bacteria, fungi, animals, and plants have been crystallized and their atomic positions resolved and reveal this enzyme complex to possess a highly conserved structure.^4,5^

**FIG. 1.**
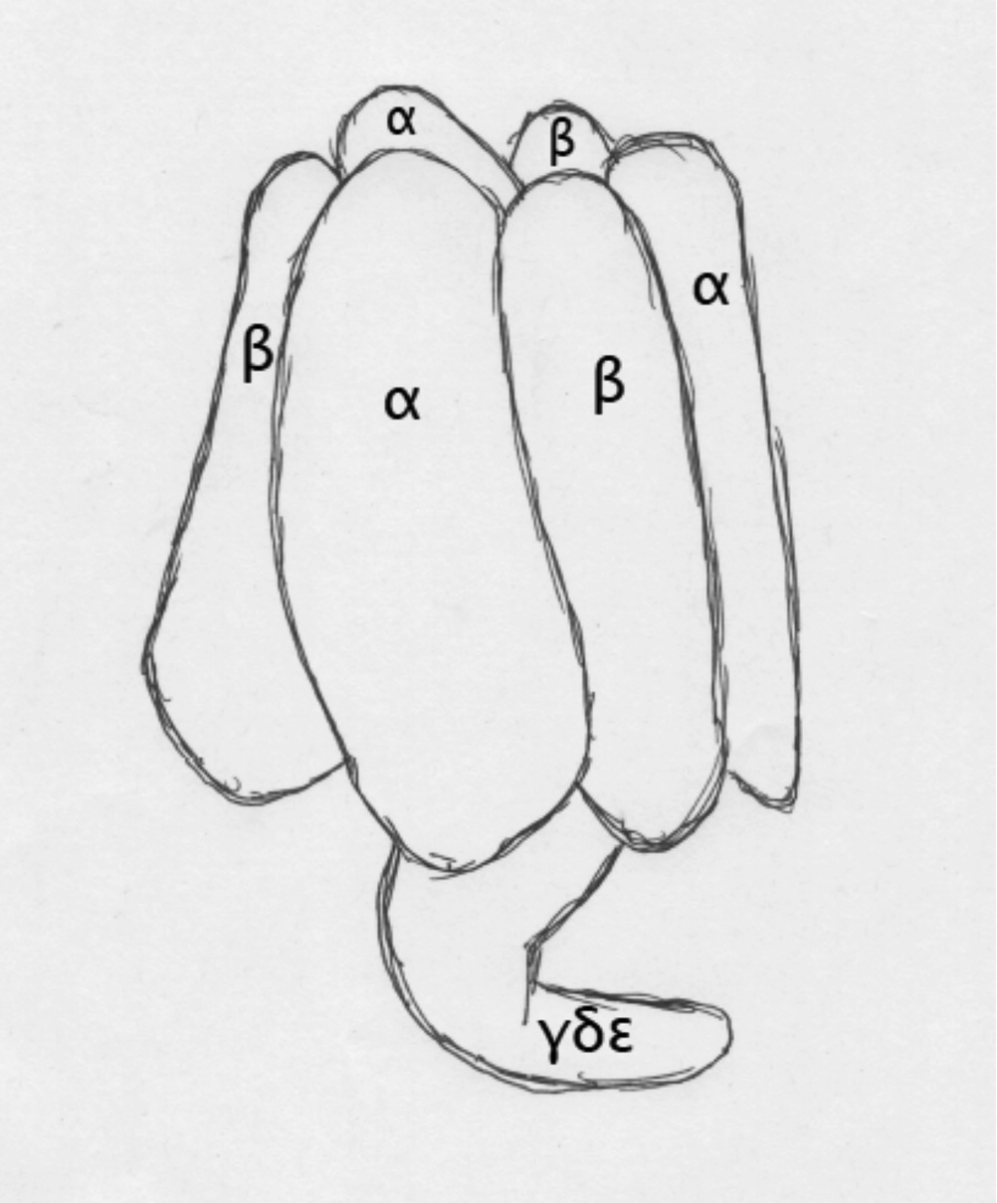
Schematic of the F_1_-ATPase fragment of ATP synthase. Composed of alternating *α* and *β* subunits, the central axis of pseudosymmetry obtains an *α*-helical coiled coil from the N and C terminal domains of the *γ* subunit, while the remainder of that subunit plus the *δ* and *ϵ* chains protrude from the central channel below the foot domains. The hexameric cap has a diameter of around 100 Å.

As illustrated in Figure 1, in F_1_-ATPase the three *α* subunits (SUA) and three *β* subunits (SUB) alternate as the segments of an orange to create a cap-like structure with an outer diameter of around 100 Å and a central channel about 20 Å across. This central channel, marking the axis of pseudosymmetry, contains a pair of coiled-coil *α* helices formed by the N and C terminal domains of the *γ* subunit. The remainder of the *γ* chain as well as the smaller *δ* and *ϵ* chains forms a globular arrangement attached to the central *α* helices like the head of a golf club to its shaft.

The X-ray structures show the *α* and *β* chains to possess nearly identical three-dimensional conformations with all-atom root mean square difference (RMSD) superpositions between 2.2 and 2.6 Å, but with primary sequence identity and similarity of 25% and 43%.^6^ Adenosyl nucleotides can bind to each SUA and SUB in binding pockets located at their interfaces. However, only SUB is catalytically active: ATP bound to SUA is neither hydrolyzed nor exchanged with solvent medium.^7–9^ Catalysis at the three *β* subunits occurs not with use of high energy intermediates but in a cooperative, cyclic fashion termed the binding change mechanism.^10^ Studying heavy oxygen exchange rates during ATP synthase catalysis in the presence and absence of a proton gradient, Boyer realized that the *pmf* at F_*o*_ is energetically coupled with product release at F_1_ rather than chemical bond-formation. Once bound to a catalytic site, in other words, ADP and P_*i*_ spontaneously interconvert to ATP without external energy and have an equilibrium constant close to 1. According to the binding change mechanism, each *β* subunit sequentially binds ADP and P_*i*_, then undergoes a conformational transition and makes ATP, and finally changes conformation again with the release of the product. The three subunits function in concert, with each subunit cycling through the same states consecutively, so that the system “hangs” if one subunit is prevented from transitioning, by, for example, removal of the product from the solvent medium. Experiments suggest that the rotary, cyclic behavior of the hexamer persists during hydrolysis in the absence of the *γ* chain, though with lower precision and rate constants.^11–13^

Our current analyses will focus on the elements comprising this minimal functional unit, the *α* and *β* chains. In particular, we examine the question: why do *β* subunits readily hydrolyze ATP and exchange the HOH generated with medium water, while the *α* subunits neither hydrolyze nor exchange ATP with solvent nucleotides? Xu and coworkers^1^ point out that while the nucleotide-binding sites in *α* and *β* subunits are closely conserved, one carboxylate of residue *β*-Glu 188 is replaced by *α*-Gln 208, eliminating a likely catalytic base in the *α* subunits. Furthermore, Xu points out that the *α* subunit's “inability to transition between different catalytic conformations, as evidenced by the absence of open conformation” in crystalline structures, severely dampens their catalytic activity. In this work, we closely examine the extent and reason for the “inability” of *α* subunits to cycle through the conformations adopted by the *β* subunits. Superficially, one might expect two proteins with such similar fold and architecture to exhibit similar flexibility characteristics. In particular, then, the internal symmetry axes of these different protein chains should be nearly identical. Are they? We compute and examine each protein's slowest eigenmodes via PDB-NMA (Protein Data Bank structure Normal Mode Analysis). We observe interesting similarities and differences that may help further explain the different catalytic propensities of these two subunits.

### B. Overview of PDB-NMA

Just as rigid structures obtain 3 rotational principal symmetry axes determined by diagonalization of their inertia matrix, nonrigid objects obtain internal symmetry axes derived from diagonalization of their Hessian matrix.^14^ An object has as many internal symmetry axes or eigenvectors as it has internal degrees of freedom, with each eigenvector a specific linear combination of those internal degrees of freedom. The Hessian matrix of an object describes the distribution, not of masses, but of forces about a position of stability as its internal degrees of freedom are varied. Rigid principal axes pertain to spatial symmetries where the mass distributions about each of 3 special, orthogonal axes are balanced and the angular velocity and momentum vectors aligned. Nonrigid, internal axes pertain to temporal symmetries: each axis pertains to a balanced, internal oscillation of the object with one particular frequency and energy. These axes are orthogonal and hence also known as normal modes: excitation of one mode cannot excite another mode, though in practice anharmonicities introduce off-diagonal elements that decohere motion. As the normal modes describe oscillations innate or intrinsic to the system, the modes are often referred to as eigenmodes and they form a complete, orthonormal coordinate system.

The set of frequencies obtained by diagonalization of the object's Hessian, called the eigenspectrum, has a characteristic distribution for proteins, different from other solids.^15–17^ The highest frequencies arise from rapid vibrations of the stiffest elements while the slowest frequency is a function of the mass and shape of the molecule and is roughly 0.1 THz for a 50 kDa protein (spectroscopists attempt to probe such motions by delivering photons with identical frequencies and often report this in terms of the corresponding photon wavenumber of 3 cm^−1^).

In addition to the eigenfrequencies, the diagonalization of the Hessian matrix provides the set of vectors or eigenmodes that describe the shape of the motion associated with each eigenfrequency. As folded, globular proteins obtain high packing densities and heat capacities comparable to solid crystalline objects,^18^ it is interesting to ascertain how correlated motions extending over the entire molecule are enabled. Snapshots of single conformations cannot convey this information and one cannot anticipate how a particular molecule “solves” the problem of enabling large-scale motions where thousands (internal) degrees of freedom cooperatively deform tens of thousands of nonbonded interactions (NBIs) to achieve correlated motions across the entire molecule or “full body motion,” FBM.

The innate *dof*s associated with equilibrium oscillations describe motilities of interest: energy imparted to an object at rest will be dissipated by these internal degrees of freedom. Unlike spectroscopists who deliver very precise energy pulses that may match particular molecular frequencies,^19^ thermal baths activate folded protein's many modes equally. No particular *dof* dissipates the thermal energy; instead, the heat energy is distributed amongst all the internal symmetry axes. The high frequency modes dissipate the kT thermal units in high frequency, low amplitude oscillations while the slowest modes dissipate kT energy units in low frequency, larger amplitude oscillations. There is a longstanding assumption that nature exploits the long-correlation length “slow” motions intrinsic in folded proteins to achieve functionality.^20^

### C. Range of validity

Normal modes provide another “quality” or signature of an object much as do mass or charge distributions, heat capacity, reflectivity, shape, etc., independent of the basis vectors chosen to describe that object's internal degrees of freedom.^21^ For this reason, the modes computed using all-atom Cartesian degrees of freedom or heavy-atom dihedral degrees of freedom or reduced coordinates such as a single point per residue match for sufficiently slow frequency modes.

All-atom analyses on PDB entries using detailed force fields such as GROMACS (GRoningen MAchine for Chemical Simulations) or CHARMM (Chemistry at HARvard Macromolecular Mechanics) are accurate but handicapped by the fact that PDB entries are not at equilibrium according to these parameterizations.^22^ Therefore, prior to diagonalization of the Hessian, an initial structure-distorting energy minimization *must* be performed. Minimization of objects with thousands of degrees of freedom and tens or hundreds of thousands of energy terms is not an exact, analytic process, and no single, unique minimized structure exists, even under a particular force field. Furthermore, the tens of thousands of nonbonded terms have exquisitely sensitive dependencies on their distances of separation that result in the rapid accumulation of round-off errors for the floating point representations of the energy per conformation. In practice, this means that a minimal energy conformation cannot be discerned using double precision computations for proteins larger than around 150 residues. The resulting negative eigenvalues describe unstable motions that are typically ignored and that complicate the analysis of the remaining positive, stable eigenmodes. One can avoid these limitations by accepting each PDB entry as representing a stable conformation, a not unreasonable assumption given that typically 10^17^ molecules align identically to provide high resolution X-ray diffraction data.^23^ This assumption, that the PDB entry represents a stable, long-lived conformation, permits design of simplified, Hookean force fields to describe equilibrium oscillations.^24^ While initial parameterizations of such Hookean force fields were necessarily maximally simple, current formulations carefully parameterize every bonded and nonbonded term in accordance with a “parent” potential such as ENCAD (ENergy CAlculation and Dynamics) or CHARMM.^25,26^ The resultant eigenspectra and eigenmodes reproduce those obtained using the parent potentials *when done on the same, energy minimized coordinates*. Consequently, the use of these Hookean force fields permits comparison of the eigenspectra and eigenmodes of two closely similar structures, such as the A and B populations within a single protein crystal^27^ or the *α* and *β* chains of F_1_-ATPase.

Our current analyses are based on a reduced heavy-atom representation, including all atoms in the PDB entries, as provided by the force field developed by Michael Levitt, termed L79,^28^ the precursor to ENCAD.^29^ In L79, the energy of each pair of (ϕ,ψ) main chain torsions is modeled as a sum of Gaussian-like potentials, a functional that reproduces Ramachandran maps and compensates for the exclusion of nonbonded interactions between atoms separated by three bonds; while the energy associated with side chain torsions is modeled by a harmonic functional. Nonbonded, dispersive, and repulsive forces between all atoms more than three bond lengths and less than some cutoff distance apart are modeled by a Lennard-Jones 6–12 potential. L79 includes only polar hydrogens, with hydrogens bonded to carbons combined into united forms.

This early formulation of an intra-molecular force field works well for modeling the motility of large, isolated globular proteins. To better characterize surface forces involved in ligand binding, inter-molecular interactions and water, especially to reaction pathways, more sophisticated potentials evolved. Our current interest pertains to analyses of the entire molecule, full-body motions, FBM, that examines the concurrent activation of thousands of internal *dof*s that cooperatively deform tens of thousands of NBI. While a Hookean force field has a seemingly trivial complexity and range of validity (namely, at a point of equilibrium), the space where several thousand constrained degrees of freedom (dihedral angles) exist in a force field consisting of tens of thousands of nonbonded pairwise interactions is rich and surprising. Achievement of self-consistency in this vast space, both in terms of the mathematical formulations and machine encoding, is essential to link effects and causes. The addition of complexity to this formulation, by inclusion of all hydrogen atoms and crystal and solvent waters, for example, is desirable, but their contributions to the FBM can only be discerned and ultimately understood in contrast to the formulation lacking these elements. For this reason, we characterize the equilibrium motility spectra of PDB entries primarily using the simpler L79 potential, and only briefly consider the contributions within the current formulation of ligands, including nucleotides, cations, and crystal HOH.

Normal mode studies of the F_1_-ATPase structure with the PDB designation^30^ 1BMF^31^ have been published by Cui and coworkers^32^ as well as by Zheng.^33^ The latter uses a coarse-grained elastic network model to study the coupling of cyclic conformational transitions, as modeled by intramonomer hinges and intermonomer rigidity body motions, and *γ* subunit rotations to ATP binding and product release. The former, earlier, study used all heavy atoms as well as polar hydrogen atoms, ligands, and crystal waters in a classical (non-Hookean) force field in order to characterize the structural plasticity of the isolated *α*, *β,* and *γ* subunits, as well as the *α*_3_*β*_3_*γ* assembly. These analyses required initial energy minimizations that distorted the crystal coordinates by RMSD values of around 1 Å. Cui and coworkers published the root mean square fluctuations or RMSF per C_*α*_ of the intra-monomer vibrations of the *α*, *β,* and *γ* subunits, as well as for the complex. Each *β* subunit they concluded “has the functionally relevant flexibility built into its structure.” Our results reproduce the RMSF plots for the *α* and *β* subunits and indicate that the FBM patterns associated with the energy minimized 1BMF structures are largely shared by those of the current PDB entry. Our analyses add to the insights provided by the earlier studies by probing the nature and cause of the variable flexibility of the *α* and *β* subunits. We continue efforts to develop a lexicon to describe nonlocal FBM where the motility of any particular loop is causally linked with the motility of all parts of the peptide chain. Highly mobile loops, it is seen, are not mobile in isolation: regions may be stiff locally and yet obtain high temperature factors. We examine the correlations of computed and observed temperature factors and note an interesting signature suggesting the absence of one particular mode of vibration in an *α* subunit.

### D. Homunculus

**FIG. 2.**
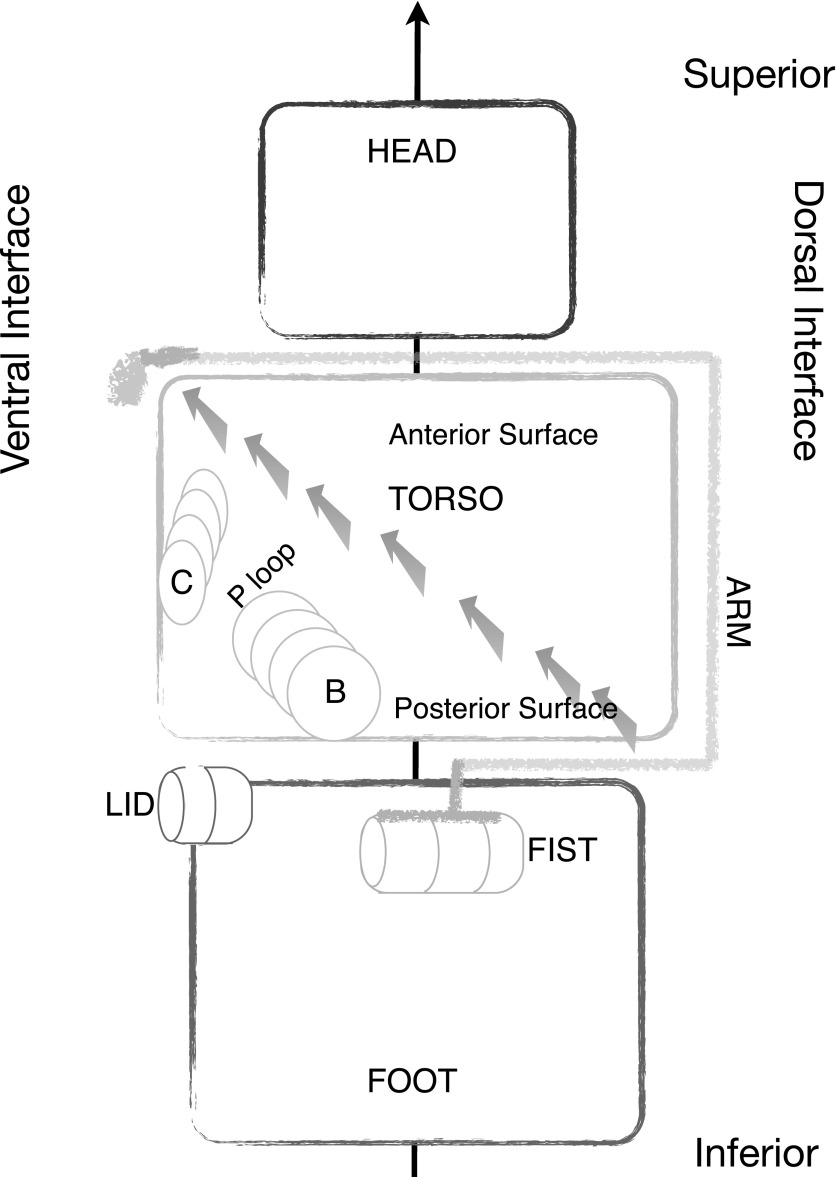
Schematic representation of the subunit. Each subunit consists of three domains, a head, torso, and foot. Viewed from the external surface, the axis of pseudosymmetry as indicated by the solid arrow is behind the subunit in this representation. Each subunit interacts with two neighbors in the hexamer: at the ventral surface and at the dorsal surface. The torso is divided into an anterior and a posterior surface region by a seven stranded parallel *β* sheet that extends from the inferior dorsal region to the superior ventral region. The posterior surface of the *β* sheet consists of the nucleotide binding region including the P-loop and helices B and C. A shoulder region at the interface of the head and torso near the ventral surface extends as an arm along the anterior surface to connect to the foot domain as a short *α* helix, the fist.

To characterize the nonlocal FBM, it will be helpful to adopt and extend a descriptive vocabulary. Figure 2 presents a schematic diagram of one subunit to provide a contextual lexicon for the regions relevant to FBM. The N terminal “head” domain, a six-stranded *β* barrel extending from the N terminus to residue *β* Asp 77 and *α* Ile 94, is *superior* to the “torso,” the nucleotide-binding domain consisting of a seven-stranded parallel *β* sheet and associated *α* helices. The C-terminal “foot” domain, a bundle of 6 (*β* subunit) or 7 (*α* subunit) *α* helices, is *inferior* to the torso in this representation. Each subunit forms two interfaces within the hexamer: with the central, rotor axis situated *internal* to the subunit, a *ventral* surface involving the subunit's nucleotide-binding domain and a *dorsal* surface that abuts the neighboring subunit's nucleotide-binding region are indicated in Figure 2.

The torso's central *β* sheet extends diagonally from the inferior dorsal region to the superior ventral region. The C-terminal, arrowed ends of the *β* strands orient towards the internal surface of the hexamer, at a lower radial distance from the central axis than the N terminal ends of the *β* strands which orient towards the *external* surface of the hexamer. The *anterior* surface of the torso's *β* sheet faces the head region and obtains 4 *α* helices and associated loops. The *posterior* surface of the *β* sheet faces the foot and obtains two *α* helices (B and C) as well as the P-loop that binds that nucleotide and cation.

The head domain links to the torso domain via a short “neck.” A long linker “arm” extending from residues *β* Asp 103-Ile 137 and *α* Asp 116-Ile 150 drapes along the outer surface of the torso from the head region to the foot region where it connects to a firmly embedded short *α* helix, the “fist,” at residues *β* 138–143 and *α* 151–155. A “shoulder” region extending from the neck to the arm is situated superior to the torso at the ventral surface. The superior, ventral region of the foot domain contributes either a highly mobile “lid” (*β* Pro 417–Pro 433) or a shorter “strut” (*α* Gln 430–Glu 440) that serve to either enhance or dampen oscillatory motions at the nucleotide-binding cleft.

## II. RESULTS

### A. PDB coordinates

We use the *α* and *β* chains from coordinate file 2JDI.^34^ For this structure, mitochondrial F_1_-ATPase from bovine heart tissue was crystallized in the absence of preservatives and inhibitors and in the presence of ADP and a non-hydrolyzable ATP analog AMP-PNP (abbreviated as ANP) and solved to 1.9 Å resolution. The 3 *α* subunits, chains A, B, and C, as well as two *β* chains, D and F, each contain ANP and cation in their nucleotide-binding pockets while the remaining *β* subunit, chain E, contains no nucleotide nor cation. Each chain folds to fit inside a wedge about 85 Å along the central axis, 50 Å in the radial direction, and 55 Å along the radial arc. By convention, each *β* subunit is paired with the neighboring *α* subunit that abuts and contributes to that *β* subunit's catalytic site: E with A, F with B, and D with C.

In 2JDI the three *α* chains adopt almost identical conformations. Their all-atom superpositions result in RMSD values of 0.6–0.7 Å, with minor mismatches in the alignment of either their head or foot regions. The *β* chains D and F, both containing ANP and Mg, obtain an RMSD of 0.6 Å. Chain E, lacking nucleotide and cation, differs from the other two *β* chains by 3.8 Å. The E chain's head and foot regions have swung to a higher radial distance from the central axis, creating a more open or extended conformation. Superposing the individual head, torso, and foot domains of chains E and F result in RMSD values of 0.2 Å, 1.3 Å, and 0.5 Å, indicating that the nearly 4 Å shift between these two chains is created to a large extent by rigid body rotations of these three domains.

The *α* chains extend from residues 24–510 and the *β* chains from 9–474 (we maintain the PDB numbering convention that labels the N terminal *β*Ala residue as 1, not-4). Chain C, as an example, obtains 3715 atomic coordinates, whose 11 145 internal, Cartesian degrees of freedom divide among 3766 bond lengths, 4768 bond angles, 2605 dihedral angles, and 6 rigid body degrees of freedom. We include only the “soft” dihedrals, including 469 ϕ, 487 *ψ*, and 873 *χ* angles, to study thermally induced equilibrium vibrations, reducing the available degrees of freedom from 11 139 to 1829 while maintaining all bond lengths and angles to PDB values. In addition to the energies associated with these *dof*s, chain C obtains 22 160 nonbonded interactions (NBIs) between all atom pairs further apart than 3 bond lengths and less than a cutoff distance defined by the inflection point of their van der Waal curves. The average NBI per atom is 5.3 and includes roughly the first shell of neighbors. The total NBI divides between 9461 main chain-main chain interactions, 9222 main chain-side chain interactions, and 3477 side chain-side chain interactions. While we report on this distribution of dihedral angles and NBI, one might examine the effects of eliminating particular groups of NBI or soft dihedrals on the motility spectra, to assess, for example, the effects of mutations on motility.^27^ (In the isolated protein, the two different orientations of surface SC Arg 373 from chain A do not affect the slow modes here described.)

### B. Normal modes

Normal modes were computed using ATMAN.^26^ Thermal activations of modes were computed at 180 K, the temperature observed to divide harmonic from anharmonic motion in folded proteins^35^ and close to the crystal temperature of 100 K. RMSF values per mode decrease rapidly with mode number, with the first three modes contributing 64% to the total; therefore, our focus remains on these three softest, slowest modes.^36^ Most analyses were carried out on chains C and F to study the distinct signatures of SUA and SUB. Residue to residue comparisons between chains belonging to subsets *α* and *β* used the Needleman and Wunsch sequence alignment algorithm available from the Protein Data Bank.^6^ Results were checked for consistency using chains D and A. Chains B and E both miss an 8 residue sequence in the foot (*α* 402–409, *β* 388–395) in a region with high experimental temperature factors (over 60 Å^2^ when the mean B-factor for chains A-F is 16 Å^2^). To test for consistency with chains B and E, the missing 8 residues were built-in by rigid body alignment of the missing region from a neighboring subunit.

**FIG. 3.**
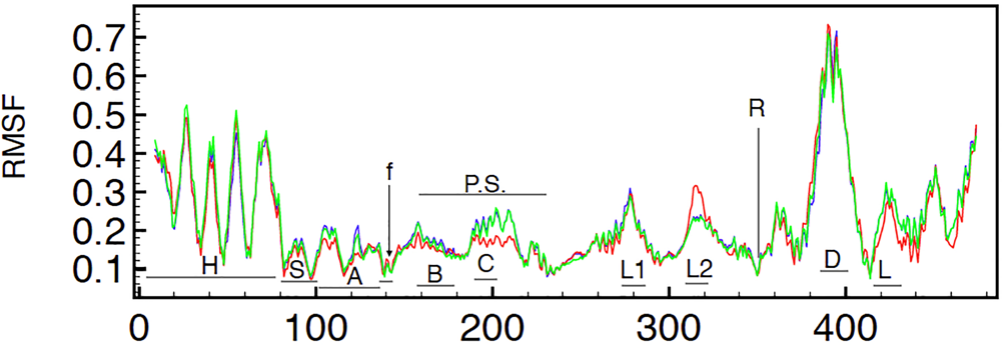
The computed RMSF in Å per C_*α*_ due to the combined contributions of modes 1, 2, and 3 at 180 K for chains D (blue), E (red), and F (green). Horizontal bars identify regions: H head; S shoulder; A arm; f fist; B and C helix B and C; P.S. posterior surface of the torso's *β* sheet; L1 loop *β*Pro 276–Pro 283; L2 loop *β*Pro 313–Pro 322; R identifies location of the Arginine finger (*β*356) that separates the torso and foot domains; D residues 390–400 include the mobile DELSEED region; L the mobile lid (*β* Pro 417–Pro 433) that forms the posterior surface of the nucleotide binding cleft. Interestingly, the regions close to and likely to interact with the *γ* rotor (D, L1, and L2) obtain relatively high mobility, even in the absence of the rotor.

In all, the computed modes describe the same motions, both among chains A, B, and C as well as among chains D, E, and F. For example, Figure 3 shows the RMSF per C_*α*_ due to the combined effects of modes 1, 2, and 3 for chains D, E, and F, the three *β* subunits. The computed motility profiles for these 3 chains superpose almost perfectly, with chain E presenting with slightly different RMSF amplitudes in the region that includes helix C (residues *β*190–215) as well as a loop on the anterior side of the torso's *β* sheet (residues *β*317–321). These plots closely match the equivalent RMSF per C_*α*_ plot for the unliganded *β* subunit, Figure 10(a), of Cui and coworkers,^32^ with the mismatch in relative amplitudes due to our use of 180 K for the activation temperature and our use of a small subset of all modes.

While the similarity of the slow modes amongst the *α* chains is not surprising due to the close structural similarity between these chains, the match of the slow modes amongst the different *β* chains is reassuring. The E chain adopts a conformation distinct from chains D and F, and the similarity of the three *β* chain mobility profiles provides a strong indication of the intrinsic character of these innate *dof*s. One might therefore expect eigenmodes to contribute to the interconversion of these different conformations.

As discussed in the Introduction, the current analyses pertain primarily to the computed eigenspectra of the *α* and *β* subunits in the absence of bound ligands. The *β* subunits transition between open and closed conformations as reactants enter and products leave the catalytic site. Furthermore, 2JDI reports 351 ± 16 crystal water molecules for the three *α* subunits, and 351 ± 85 crystal waters for the three *β* subunits, indicating a large variation in the numbers of crystal waters accompanying the structural transitions. To assess the extent to which ligands affect the expression of the slowest modes, we computed the eigenspectra and eigenvectors of the protein plus nucleotide and cation; protein plus tight bound waters; and protein plus nucleotide, cation and tight bound waters. A water molecule was considered tightly bound if it obtained at least 5 NBI with protein atoms. We find that the presence of nucleotide and cation slightly shifts the frequencies of the slowest modes, making them stiffer and resulting in slightly smaller amplitudes of oscillation at any given temperature. The presence of tightly bound waters slightly alters the slow eigenvector shapes, tending to enhance the propensity seen in the *β* subunits to open and close the catalytic site. As the effects of ligands on the slow modes as modeled by L79 do not result in significant differences, we here focus our research on the isolated protein chains.

**FIG. 4.**
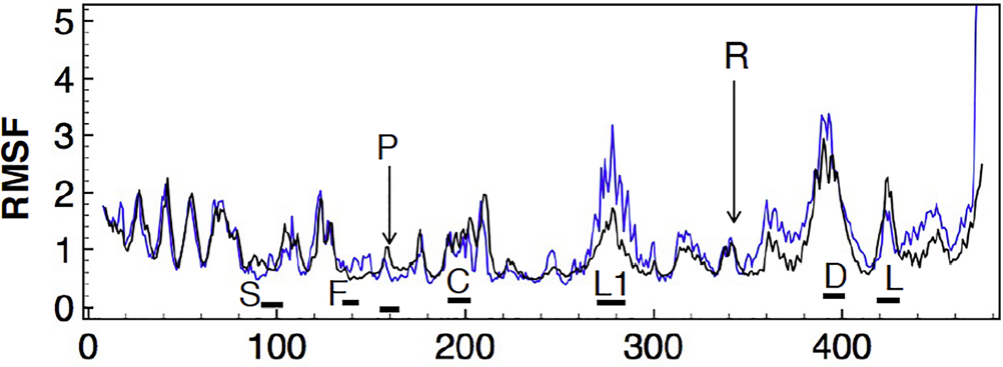
The RMSF in Å per C_*α*_ obtained from a 100 ns MD simulation of hydrated, isolated, and liganded subunit *β* (chain F) in blue^37^ and of the same chain, unliganded, computed from the slowest 50 normal modes in black. The close overlap indicates that the long term motility of the polypeptide chain is well captured by the harmonic analysis, with only slight mismatches in the magnitude of peaks associated with the fist region and P-loop region, likely due to the absence of nucleotide in the PDB-NMA; a different magnitude for the L1 peak near the *γ* rotor axis; and some mismatch in magnitude in the region immediately after the arginine finger and after the mobile lid region. Both the DELSEED loop and the mobile lid region in the foot domain overlap closely.

The use of unliganded protein chains is supported by earlier analyses of the liganded and unliganded motility profiles of *β* subunits^32,37,38^ which show RMSF per C_*α*_ shifts only in the magnitudes of certain peaks. Such shifts imply changes in the amplitudes of motions, not changes in the character of the motion. For example, Hahn-Herrera and coworkers recently published results of MD simulations of isolated *α* and *β* chains of PDB entry 2JDI.^37^ In addition to the protonated PDB coordinates, ATP and cation, his group included 40 338 water molecules in the system. After 10 ns of equilibration, the system underwent 100 ns of unbiased MD simulation. The resultant RMSF values of the C_*α*_ atoms of chain F (liganded *β* subunit) are shown as the orange line in Figure 4 (data kindly provided by Prof. Garcia-Hernandez). Current NMA predictions due to the slowest 50 modes for the same chain, unliganded and up-scaled by a factor of 2.6, are superposed with the black line. Harmonic oscillations at 300 K, rather than the 180 K used here, increase the amplitudes of oscillation by a factor of $\sqrt{300/180}$ or 1.3. Furthermore, anharmonic contributions in MD are thought to double predicted displacements compared to harmonic NMA^39^ and may explain this scaling factor. The motility patterns for the head, shoulder, arm, and posterior surface of the torso domain match closely. The peaks associated with those regions closest to the central *γ* axis, such as the DELSEED loop, are also matched, though with slight mismatches in their magnitudes. By and large, therefore, the long-term, 100 ns, motility profile of a solvated protein chain derived from a detailed and accurate force field matches that predicted by PDB-NMA. This match provides a strong indication that long term motility is the result of correlated motions and not the result of separate regions moving independently. The NMA profile, produced by the superposition of all the modes of oscillation, may be separated into and studied as individual modes in an effort to characterize and apprehend the source of these long-term motility profiles.

### C. The first three modes

**FIG. 5.**
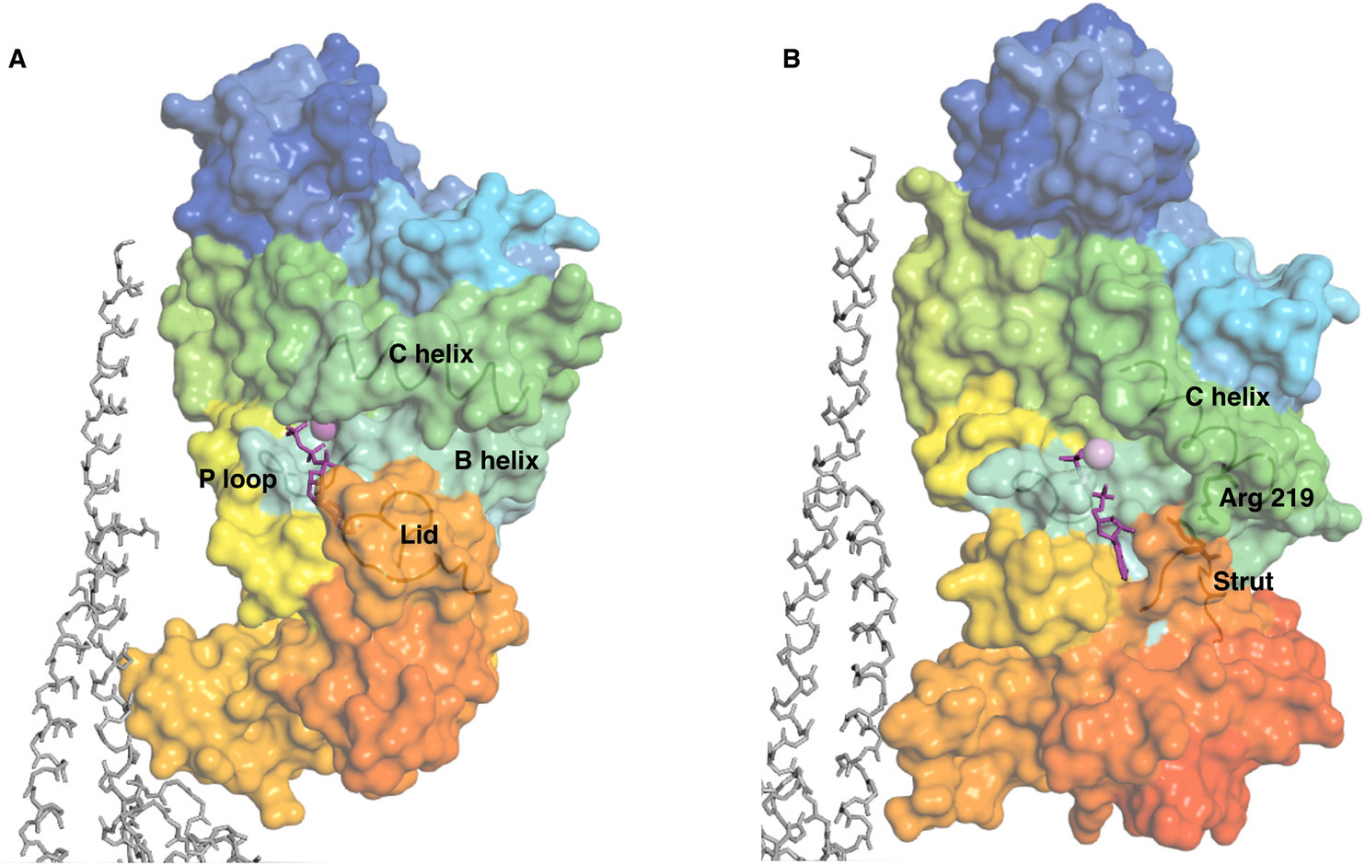
Surface representations of the ventral surfaces of SUB (left) and SUA (right) showing the nucleotide binding clefts with ATP colored magenta and Mg as a pink sphere. The N and C terminal domains of subunit *γ*'s are shown in a gray stick representation. SUA and SUB are colored with a rainbow scheme with the N terminal, head domain in dark blue, the shoulder and fist (visible beneath the strut in B) in cyan and the C terminal foot domains orange and red. The P-loop, to the left of ATP, extends as helix B in light blue and forms the base of the nucleotide binding cleft. The top of this cleft is formed by helix C and the bottom of the cleft by the mobile lid (a) or the rigid strut (b). In SUB helix C and the lid experience pronounced swinging motions in eigenmodes 1, 2, and 3, motions absent in SUA where strut residue Tyr 433 forms extensive steric interactions with helix C residue Arg 219. Figures prepared in PyMol.

The slowest three modes of SUA and SUB obtain similar profiles for the FBMs involving relative displacements of the head, torso, and foot regions. While computed using dihedral angles, the slowest three modes are also perpendicular in Cartesian space, with oscillations of mode 1 about an axis aligned along a radial direction, of mode 2 about a radial arc, and for mode 3 about a direction parallel to the central, *γ* axis. While the oscillations described by each mode appear largely similar between SUA and SUB, their effects on the posterior side of the *β* sheet, and in particular, on the cleft that ends at the Walker A motif or P-loop (GxxxxGKT, residues *α* 169–177 and *β* 156–163) that coordinates the *β* phosphate of the nucleotide, are very distinct. In SUB this cleft extends approximately 20 Å along a radial direction to the external surface with its base formed by helix B extending from the P-loop (Figure 5(a)). The top of the cleft is formed by helix C and the bottom by the lid, with *β*Phe 424 aligned with the ribose ring. This cleft in SUB displays pronounced opening and closing motions for modes 1 and 2 and a relative, grinding motion in mode 3, motions completely absent in the *α* subunits. The mobile lid is not present in SUA where the strut blocks relative motion across the crevasse, with foot residue *α*Tyr 433 forming extensive steric interactions with *α* Arg 219 from the top of the cleft (Figure 5(b)).

**FIG. 6.**
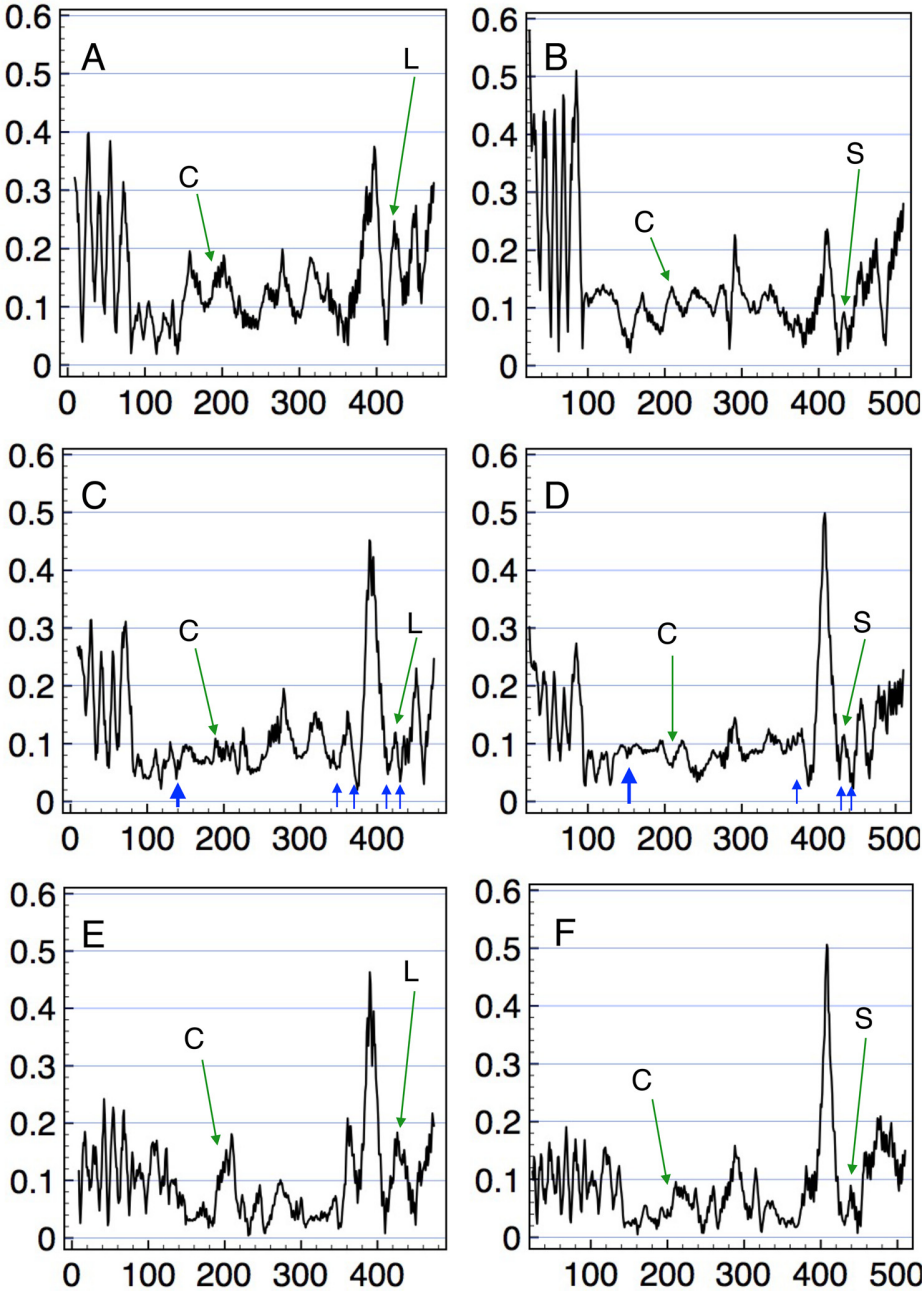
RMSF in Å per C_*α*_ for mode 1 (a) and (b), mode 2 (c) and (d), and mode 3 (e) and (f) with left images for SUB and right images for SUA. Peaks associated with helix C and either the lid L (SUB) or the strut S (SUA) is indicated by slanted green arrows. The short vertical blue arrows indicate regions of low motility near to the fist residues.

The slowest modes of SUA and SUB (Figures 6(a) and 6(b)), with frequencies of *α* 2.6 cm^−1^ and *β* 2.7 cm^−1^, pertain to a sidewise rolling, with the head and foot regions rocking towards each other along a radial axis closely aligned with the P-loop helix (B). The arm in SUB seems to function as a caliper, with the *C_α_*s of shoulder residues Asp 103 and fist Gly 136 separating 33.2 ± 1.2 Å. This results, on the other side of the torso's *β* sheet, in a sizable fluctuation of nearly 3 Å between the top of the cleft and the mobile lid, in an up-and-down chewing type motion. In SUA, the rolling of the head towards and away from the foot along a radial axis is again observed. However, the arm in SUA does not function as a caliper, and the equivalent *C_α_* atoms at residues Asp 116 and Gly 149 remain at a nearly steady 28.5 ± 0.2 Å separation during thermal activation. As a result, the cleft and access to the cleft leading to the P-loop is not distorted.

Modes 2 (Figures 6(c) and 6(d)), both with a frequency of 3.1 cm^−1^, pertain to a flexing toward the central axis, with a short stationary *α* helix oriented along a radial arc marking the axis about which the top and bottom portions of the chain oscillate. This short helix is the fist that interconnects the head and foot regions, moving in tight synchrony with the foot domain due to extensive packing interactions, including SUB residues Lys 138, Val 139, Leu 142, and Leu 143. The NZ of Lys 138, for example, maintains fixed distances of separation with the main chain carbonyl oxygens of Arg 142, Phe 457, Gly 461, Phe 413, and Val 460 during thermal activation. The effect, as seen in Figure 6(c), is startling: the fist residues *β* 138–143 (indicated by the short, left-most vertical arrow) obtain low RMSF values along with those nearby regions of the foot indicated by the remaining vertical arrows. Several of the *α* helices in the foot radiate away from the fist region, with those regions of the helices near the fist nearly immobile while the remainder of these helices obtain significant RMSF values. For example, the C terminal region of helix *β*399–414 and the N terminal region of helix *β*434–448 obtain small RMSF values (right most arrows), while their connecting loop forms the mobile lid motif. As a result, the head and foot regions simultaneously rock towards and away from the central *γ* axis, with the nucleotide binding cleft experiencing a more pronounced opening nearer the P-loop rather than near the outer edge as in mode 1. The second mode, as will be seen, contributes the largest amplitude when the slow modes are used as *dof*s to transform the coordinates of the D and F chain to the E chain.

Modes 3 (Figures 6(e) and 6(f)), with computed frequencies of *α* 3.8 cm^−1^ and *β* 3.6 cm^−1^, pertain to a twisting motion of the head and foot regions about an axis roughly parallel to the central *γ* axis. This motion reveals considerable swinging of that portion of the foot that includes the DELSEED region nearest the central *γ* axis and is coupled to an appreciable relative twisting of the top and bottom of the nucleotide binding cleft in SUB. As an example, the 11.2 Å distance of separation in SUB between lid atom Phe 424 CZ and Arg 189 CZ at the N terminal end of helix C reduces to 10.4 Å before increasing to 12.3 Å during one cycle of oscillation, while the 16.5 Å distance of separation between the same Phe 424 CZ and Glu 202 CD at the C terminal end of helix C, first increases to 18.0 Å before reducing to 15.0 Å during the same cycle of oscillation. This motion seems enabled by the torso's central *β* sheet as the twist angle between adjacent strands of the *β* sheet varies slightly as the head and foot domains rotate in opposite sense.^40^ As before, this distortion of the nucleotide binding cleft is not seen in SUA.

We next examine the correlations of the computed motilities against the experimentally determined temperature factors before computing to what extent these three orthogonal modes reduce the RMSD between the closed and open *β* monomers. In the discussion, we will consider the sources of the variable stiffness characteristics in SUA and SUB.

### D. Correlations with crystallographic temperature factors

**FIG. 7.**
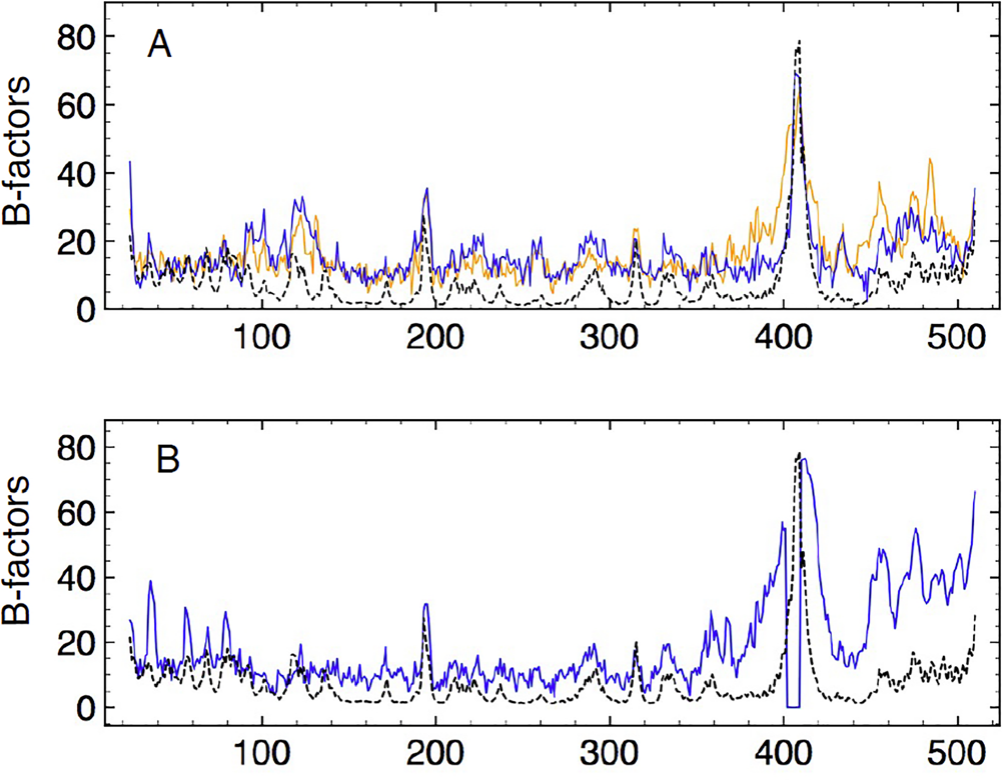
B-factors in Å^2^ per C_*α*_ for the *α* subunits: experimental data for chains A and C in orange and blue in top panel and for chain B in blue, bottom panel. The PDB entry for chain B misses residues 402–409. Superposed with the broken black line, the unscaled computed B-factors derived from the sum of modes 2–50, top panel, and modes 1–50, bottom panel. The cross correlations of the theoretical and experimental B-factors for chain C are 0.68 (modes 1–50) and 0.80 (modes 2–50). In contrast, the cross correlations for chain B are 0.58 (modes 1–50) and 0.59 (modes 2–50), demonstrating that the experimental B-factors for chains A and C, but not for chain B with its distinct head peaks, are better reproduced by excluding mode 1 in the sum.

We were interested to see how well the crystallographically determined Debye-Waller or B-factors for the three *α* chains as well as for the three *β* chains compared, in addition to their similarities to the computed motilities. The 3 *α* chains obtain all-atom RMSDs of around 0.7 Å, and one might expect, barring crystal packing effects, very similar B plots. In fact, the crystallographic B-factors per C_*α*_ for chains A and C are quite similar (Figure 7(a), solid lines), as expected, but chain B obtains a distinct signature (Figure 7(b), solid line), with 5 pronounced peaks in the head region, a reduced amplitude in the neck and shoulder region, and enhanced motility in the foot region. Superposed on these plots with dotted lines are the *unscaled* computed temperature factors of the first 50 modes (Figure 7(b), dashed line) and of modes 2–50 (Figure 7(a), dashed line). The experimental temperature signature of chain B is reproduced reasonably well by the contributions of the slowest 50 modes, with its distinct head peaks, helix C and DELSEED peaks as well as several other torso and foot peaks accounted for. Intriguingly, the experimental temperature factors of chains A and C are best reproduced by excluding mode 1 in the sum, which similarly eliminates the distinct peaks in the head region. One interpretation of these data is that in the 2JDI crystal, chain B vibrates along all eigenmodes while chains A and C lack the slowest degree of freedom, perhaps due to their differing interaction with the central rotor proteins. The current computations consider only intra-monomer, not inter-monomer, packing interactions, and the possibility that the source of the variable experimental Debye Waller signatures for the three *α* chains might arise from variable symmetry axes activation is a novel concept for molecules as large as proteins. Laser spectroscopists have successfully activated single resonances (eigenfrequencies) in multi-atom systems in order to alter reaction rates;^19,41^ perhaps the altered B chain Debye Waller factor profile in the crystal structure map is indicative of selective eigenmode damping.

**FIG. 8.**
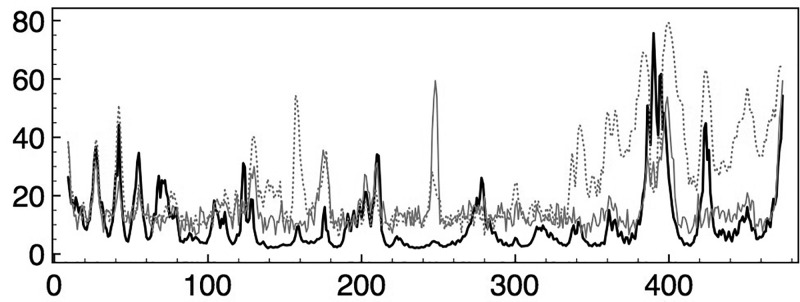
B-factors in Å^2^ per C_*α*_ for the *β* subunits: experimental data for chains D and F in solid brown and chain E in dashed brown curves. The unscaled theoretical values are superposed in solid black. The cross correlations of the theoretical and experimental B-factors are 0.56 (chains D and F) and 0.32 (chain E). The experimental B-factor data for chains D and F are reasonably well modeled by PDB-NMA; the data for chain E less well. Each crystalline hexamer unit obtains one unliganded *β* subunit (chain E) with altered solvation characteristics due to its open configuration. Reduced statistics combined with possible greater variability in the conformation of the foot domain, including the fist, might explain the mismatch. The peak at 245 corresponds to a loop on the anterior surface of the torso's *β* sheet, at high radius, and is involved perhaps with inter-hexamer packing interactions. The enhanced P-loop peak (156–163) motility is likely due to the absence of nucleotide and cation.

The experimental temperature factors for chains D, E, and F are shown in Figure 8. The two structurally similar subunits, D and F, display similar vibrational patterns indicated by the solid brown curve, while chain E obtains significantly higher B-factors for the C-terminal residues after the torso's final *β* strand, as well as an additional spike for those residues connecting the fist to the P-loop, as indicated by the dashed curve. Superposed on these curves in black are the computed B-factors due to the slowest 50 modes of chain F. The theoretical values reproduce some of the experimental features, obtaining similar head and foot motility profiles yet notably missing several peaks in the torso region. For example, the experimental peak at residues 238–242, a loop on the anterior surface of the torso's *β* sheet, is not predicted. As discussed, the computed motility profiles for chains D, E, and F are almost indistinguishable; hence, the source of the E chain's variable B signature is likely due to effects other than intra-minimum, equilibrium vibrations.

While experimental temperature factors include many effects other than the intra-monomer vibrations considered here, such as harmonic inter-monomer and crystalline vibrations as well as anharmonic noise, nonetheless it might be instructive to determine whether these data support the central assertion of reduced motility at the posterior surface of the torso's *β* sheet in SUA compared to SUB. PDB-NMA predicts the C_*α*_ atoms in SUA and SUB to obtain average B-factors of 9.7 Å^2^ and 10.9 Å^2^. The C_*α*_ atoms posterior to the *β* sheet, *α*169–229 and *β*157–214, obtain average theoretical B-factors of 6.2 Å^2^ and 9.9 Å^2^, indicating the reduced mobility of this region in SUA compared to SUB. The average experimental C_*α*_ B-factors for SUA and SUB are 15.8 Å^2^ and 15.9 Å^2^. The average experimental B-factors for the C_*α*_ atoms posterior to the *β* sheet are 15.5 Å^2^ and 18.8 Å^2^, indicating once again that the posterior surface of the torso's *β* sheet obtains lower motility character in SUA than in SUB. In sum, the experimental B-factors do not contradict the predictions of NMA; support the observation that the nucleotide binding region experiences reduced motility; suggests the B-factor data of the *α* subunits to be more accurately modeled by NMA than those of the *β* subunits; and may indicate selective modal damping in an *α* subunit.

### E. Interconversion of SUB structures with modes

**FIG. 9.**
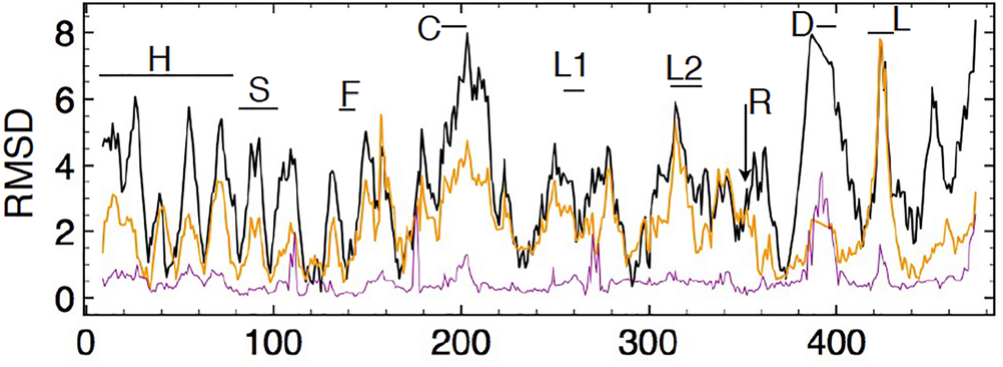
RMSD in Å per C_*α*_ between the PDB coordinates of chain E (open conformation) and chain F (closed conformation) in black, with an overall RMSD of 3.8 Å. Horizontal bars identify regions as defined in Figure 3. A similar curve for the RMSD between chain E and the normal mode deformed chain F, shown in orange, obtains an overall RMSD of 2.3 Å. For comparison, the RMSD curve for the similarly shaped closed chains D and F, with an overall RMSD of 0.6 Å, is shown in purple. These data show that the initial large mismatch in the alignment of the head, shoulder, and arm residues as well as the foot residues after the arginine finger, improves significantly after adjusting chain F coordinates along the slowest three modes. The fit of the lid (L) region does not improve, suggesting a nonharmonic contribution to the shift of this region during the interconversion of the open and closed conformations. For the torso, there is improvement in the fit in that region on the posterior surface that includes the helix C residues. The fit of the remainder of the torso region is not much improved, indicating that shifts within this region are also less well modeled by harmonic, intra-minimum oscillations exclusively. PDB 2JDI chain E lacks residues 388–395 within the DELSEED region.

By deforming the coordinates of SUB chain F along the directions of its three slowest modes, we tested to what extent those coordinates could reduce the RMSD between chains F and E (Figure 9). The initial RMSD of all mainchain heavy atoms of 3.8 Å is reduced to 2.3 Å using relative contributions of 25%, 45%, and 30% of modes 1, 2, and 3. The resultant structure shows that the transformation aligns the relative positions of the head, foot, and torso domains but does not much improve the alignments within domains, which remain at 0.25 Å, 1.2 Å, and 0.53 Å RMSD from those of chain E. For example, alignment of the crystal F and E chains shows a distance of separation of their C termini of 8.5 Å that reduces to 1.5 Å for the normal-mode-deformed F chain. Likewise, the N termini in the crystal coordinates are 4.5 Å apart, which reduces to 2.4 Å for the NM-deformed chain. Within the torso domain, however, only slight improvements to the RMSD are achieved, with observed realignment of the nucleotide binding cleft not modeled as well by the slowest three modes. The cleft separation, for example, from C helix C_*α*_ 203 to mobile lid C_*α*_ 420 in chain F is 13.8 Å and in chain E is 5.1 Å. The NM-deformed F chain reduces this to 11.1 Å.

A similar analysis of chain D reduces an initial RMSD to chain E of 3.8 Å–3.6 Å with use of 8.5 kT of mode 1, to 2.9 Å with 17.3 kT of mode 2, and to 2.7 Å with 8.5 kT of mode 3. The importance of natural or innate flexibility in effecting the open to closed transformation of *β* subunits was noted by Böckmann and Grubmüller, based on MD simulations of unliganded, open *β* subunits. They found fast, spontaneous, and nucleotide-independent closure of the open *β* subunit, with changes not localized to the nucleotide binding region and not exerted from adjacent *α* subunits: “the main driving force for the closure is internal to the *β*-subunit” they concluded.^38^

## III. DISCUSSION

### A. Design and flexibility of subunits

SUA and SUB present very similar topologies and folds leading to similar flexibility profiles. A long linker arm straddles a central torso domain to interconnect similar head and foot domains. A seven stranded parallel *β* sheet spans the torso from the ventral surface near the head to the dorsal surface near the foot and divides the torso into an anterior region involved with inter-subunit packing and a posterior region that includes the nucleotide binding cleft. Both have an important short *α* helix at the end of the linker arm embedded tightly in the foot domain. This helix forms nonbonded interactions with residues forming the torso's eighth, antiparallel *β* strand (in SUB the pattern of inter-strand hydrogen bonding precludes its designation as a *β* strand); the N terminal end of the long *α* helix after the arginine finger; the C terminal end of the foot's third *α* helix and the N terminal end of its fourth *α* helix whose connecting loop constitutes the mobile lid or the strut; as well as with the C terminal end of the torso's helix B. The fist, in other words, while still near the N terminal end of the polypeptide chain (residues 138–143), also coordinates the motion of foot residues, including the critical lid or strut, as well as torso residues. Specifically, as indicated in Figures 6(c) and 6(d), these regions, like the fist residues, obtain very small motilities, indicating the critical importance of the fist in coordinating the motions while maintaining the structural integrity of the chain's fold.

This design leads to similar motility profiles, with the softest motilities associated with a side-wise rolling, a flexing towards the central axis, and a twisting about the central axis of the head and foot domains. While the inter-domain motions of the head, foot, and torso appear largely similar between SUA and SUB, important differences exist between the motility profiles of the region posterior to the torso's *β* sheet: the nucleotide binding region. In SUB the nucleotide binding cleft exhibits pronounced opening and closing motions, motilities absent in SUA, and points to an unsurprising difference between enzymes and structural proteins. Enzymes require entry and exit of reactants and products in a manner that predicates reaction rates, whereas structural proteins have no need of such intra-domain motilities. What design features allow one chain to be an active enzyme, the other not?

### B. Source of variable torso stiffness

In SUA the residues forming the foot domain move in lockstep with the fist residues and also with all the residues comprising the posterior surface of the torso's *β* sheet. In SUB the residues forming the foot and fist move together with only helix B of the posterior surface: helix C moves *en masse* with the shoulder and residues of the anterior surface. Why? Certainly the presence in SUA of the foot's strut element and the resultant tight packing between strut residue *α*Tyr 433 and helix C residue *α*Arg 219 creates a significant obstruction to intra-cleft motilities. But in addition to this localized distinction, distributed differences exist between SUA and SUB in terms of the distribution of NBI. The 35 arm residues (*α* Asp 116-Ile 150 and *β* Asp 103–137), for example, pack more tightly in SUA, with 1186 NBI between arm atoms and non-arm atoms, and 789 such NBI in SUB. Also, as pointed out in Sec. II, the diagonal distance across the torso as measured by the distance of separation of the C_*α*_ atoms of the first and last arm residues is 33 Å in SUB and 28 Å in SUA, another indication of a more compact torso arrangement in SUA. The less tightly packed arm segment of SUB obtains two additional elements of secondary structure: a 310 helix (Phe 123–Glu 125), as well as a short antiparallel *β* sheet preceding the fist (Ile 132–Leu 133 and Tyr 146–Ala 147), both absent in SUA.

Another element that seems critical to the distinct motility profiles centers on the interface of the foot and torso domains and pertains to the fold of the chain immediately after *α* Arg 373 and *β* Arg 356. This so-called arginine finger is equivalently situated in SUA and SUB: contributed by the foot domain but oriented towards the anterior surface of the *β* sheet, at the dorsal surface between the torso and the arm. In SUA the chain after Arg 373 turns away from the dorsal interface and inserts into the cleft formed at the junction of the arm and fist where the two *β* strands preceding the fist meet. Likely this is the reason that SUA cannot form a *β* structure here: any potential *β* strand contributed by the arm segment is displaced by this loop after the arginine finger. This tight packing in SUA effectively creates a block structure where fist and foot motility transfers *en masse* to helix B and, due to the strut, to helix C. In SUB the residues after Arg 356, instead of folding away from the dorsal surface, fold towards it, creating the space that allows the formation of the locally stiff antiparallel *β* strand that precedes the fist. This arrangement eliminates the tight packed, block-like structure and creates the possibility of intra-domain motion, with helix B moving with the foot domain, and helix C moving as part of the shoulder and anterior surface of the torso.

The eigenmodes of SUA and SUB, therefore, demonstrate the two qualities that play off each other so that two chains with identical folds have different motilities: spaciousness provided by regions with relatively low density of NBI against local stiffness elements, such additional elements of secondary structure. Lack of tight packing in SUB is supplemented not only by the presence of additional rigid elements of secondary structure, but also by inclusion of additional prolines. SUB has 23 while SUA has 17 of them. Rigid body motion seems enabled by distributed, high density of NBI forcing such regions to move *en masse*. Relative motion within a domain or region requires sufficient space to enable the various elements to move in an opposing sense. Such spaciousness likely could result in localized fraying or unfolding of the polypeptide chain, where it is not offset by these elements of local stiffness. In both cases, there is effective and fine-tuned transmission of stiffness throughout the chain. The distributed nature of the “control” of mobility characteristics minimizes likelihood of disruption but also permits higher precision. For example, the absence of the strut element in SUB permits motility within the nucleotide binding cleft, but this additional degree of freedom is not disorderly or haphazard: many nonlocalized, distributed elements conspire to control the expression of this degree of freedom.

### C. Intra-monomer oscillations within assembly

Eigenmodes were computed for isolated *α* and *β* chains without regard to the additional inter-monomer NBIs in the assembly; hence, steric clashes were expected when these intra-monomer modes were activated within the assembly. Unexpectedly, steric inter-monomer clashes are avoided in the assembly when the isolated-monomer modes were activated simultaneously. The interfaces maintain their interdigitation during activation of each (intra-monomer) mode, with the projections of one subunit maintaining closely similar dispositions with the concavities of its neighbor. This effect seems to be caused by the relative immobility of those residues involved with inter-monomer interactions.

In particular, a ring of connectivity that extends around the assembly at the level of the superior surface of the torso maintains close interdigitations, with shoulder elements of one subunit packing with arm elements of the neighboring subunit. Specifically, at the dorsal surface of SUA, arm residues *α* Pro 134–Pro 138 form a loop construct that latch tightly with the neighbor's shoulder loop *β* Pro 101–Pro 107, with *α* Ile 136–Ile 137 buttressed between the neighbor's shoulder loop and the N terminal (inner) end of its helix C. Both interdigitating loops at this dorsal surface are braced by proline residues that provide the requisite rigidity to these latch elements.

At the ventral surface of SUA, meanwhile, shoulder loop residues *α* Ala 114–Pro 120 form the loop construct that together with helix C latch onto the neighbor's *β* Pro 121–Glu 125 arm residues, with the protrusion of *β* Phe 123 maintaining a tightly coordinated orientation with the neighboring subunit during thermal activation. Note that one of the two bracing prolines in the SUB's arm loop is replaced by a short 310 helix at residues *β* Phe 123–Glu 125 that seems to confer the requisite rigidity to help maintain hexamer integrity.

Activating the oscillatory motion of each mode simultaneously therefore suggests a relatively immobile anterior torso surface coordinating hexamer stability while the foot and posterior torso surface seem “free” to oscillate independently of its neighbors. This feature of isolated individual components comprising an assembly already possessing the flexibility characteristics suitable for the ensemble and not imposed by the assembly is surprising and indicative of an active two-way selection pressure from the ground up as well as from the top down.^42^ Initial assumptions, that the rotary catalysis mechanism of ATP synthase is governed by the rotation of the central *γδϵ* unit that forces each SUB into different conformations, ignores these intrinsic propensities that help explain the observation of rotary catalysis F in rotorless hexamers.

## IV. CONCLUSION

We examined the reasons why two proteins with nearly identical topology and fold but with different primary sequences behave very differently within the same assembly: one as a structural protein and the other as an enzyme. Without distorting the PDB coordinates, we computed the distinct symmetry axes of each protein via PDB-NMA and discovered unique signatures that help explain their differing behaviors. PDB-NMA consistently demonstrates the intrinsic flexibility around active sites in enzymes where reactants and reaction products enter and exit. Structural proteins like SUA do not develop the flexibility characteristics associated with interdomain motilities, where similarly disposed regions move, more simply, with block-like character. Active site motility is enabled by relatively low NBI or packing densities in critical junctures that create sufficient space to permit opening and closing movements. Any resultant structural weakness is offset by local stiffness elements, including *β* sheets, helices, and prolines.

In addition to developing insights into the particular three-dimensional architecture of a stably folded protein, PDB-NMA demonstrates how locally stiff regions may obtain very high temperature factors. Residues with high experimental Debye-Waller factors likely are not disordered, but orderedly mobile, an observation supported not only by crystallographic data but also by MD simulations that observe similar long term behavior as NMA. Enzymes function as machines that are firm and flexible, and with flexibility characteristics that are reliable and reproducible.

We deformed the coordinates of a closed *β* chain along the directions of its slowest three modes to achieve better overlap with the coordinates of the open chain. An initial RMSD of 4 Å between these structures was thereby reduced to 2 Å, without creating steric clashes. The resultant structure achieves close inter-domain fits of the head, foot, and torso and less close fits for intra-domain shifts, especially of the nucleotide binding region. During the structural transformation of the *β* subunit, this region undergoes major shifts in solvation, a feature not modeled by the current analysis and likely a major reason for the poorer fit. Finally, PDB-NMA permits examination of observed and computed temperature factors to search for clues in mobility profiles of different chains. In the case of the three *α* subunits in 2JDI, two distinct B-factor profiles were observed that might be indicative of modal suppression of the slowest mode in these two chains.
